# *N*,*N*′-Bis-(2,4-dichloro­benzyl­idene)-2,2-dimethyl­propane-1,3-diamine

**DOI:** 10.1107/S1600536808035745

**Published:** 2008-11-08

**Authors:** Reza Kia, Hoong-Kun Fun, Hadi Kargar

**Affiliations:** a X-ray Crystallography Unit, School of Physics, Universiti Sains Malaysia, 11800 USM, Penang, Malaysia; b Department of Chemistry, School of Science, Payame Noor University (PNU), Ardakan, Yazd, Iran

## Abstract

The mol­ecule of the title Schiff base compound, C_19_H_18_Cl_4_N_2_, has crystallographic twofold rotation symmetry, with one C atom lying on the rotation axis. The dihedral angle between the two symmetry-related benzene rings is 84.70 (2)°. The plane of the –C=N—C– group is twisted away from the benzene ring by 7.5 (1)°. In the crystal structure, weak inter­molecular Cl⋯Cl [3.4851 (3) Å] contacts link neighbouring mol­ecules into a two-dimensional network parallel to the *bc* plane.

## Related literature

For bond-length data, see: Allen *et al.* (1987 ▶). For related structures, see: Li *et al.* (2005 ▶); Bomfim *et al.* (2005 ▶); Glidewell *et al.* (2005 ▶, 2006 ▶); Sun *et al.* (2004 ▶); Fun *et al.* (2008 ▶).
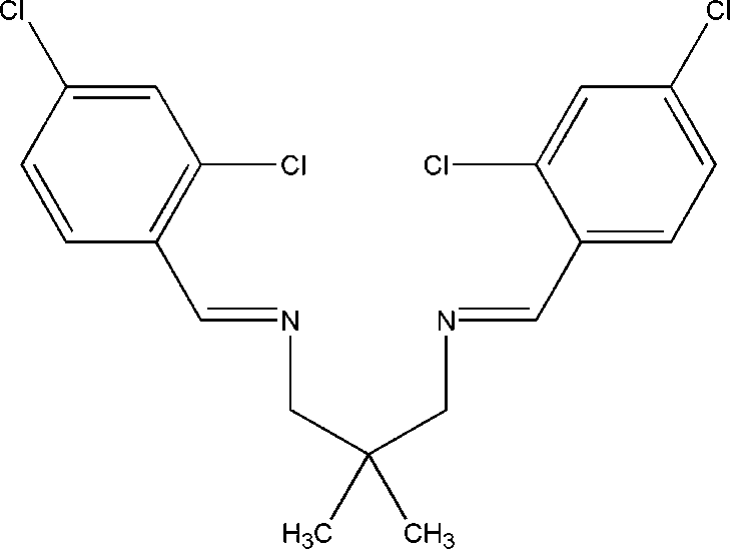


## Experimental

### 

#### Crystal data

C_19_H_18_Cl_4_N_2_
*M* *_r_* = 416.15
Orthorhombic, 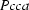

*a* = 30.7633 (4) Å
*b* = 5.4012 (1) Å
*c* = 11.4532 (1) Å
*V* = 1903.05 (5) Å^3^
*Z* = 4
Mo *K*α radiation
μ = 0.63 mm^−1^
*T* = 100.0 (1) K
0.43 × 0.25 × 0.23 mm

#### Data collection

Bruker SMART APEXII CCD area-detector diffractometer
Absorption correction: multi-scan (*SADABS*; Bruker, 2005 ▶) *T*_min_ = 0.774, *T*_max_ = 0.872
86683 measured reflections
6565 independent reflections
5678 reflections with *I* > 2σ(*I*)
*R*_int_ = 0.028

#### Refinement

*R*[*F* ^2^ > 2σ(*F* ^2^)] = 0.030
*wR*(*F* ^2^) = 0.099
*S* = 1.19
4992 reflections
115 parameters
H-atom parameters constrained
Δρ_max_ = 0.51 e Å^−3^
Δρ_min_ = −0.30 e Å^−3^

### 

Data collection: *APEX2* (Bruker, 2005 ▶); cell refinement: *SAINT* (Bruker, 2005 ▶); data reduction: *SAINT*; program(s) used to solve structure: *SHELXTL* (Sheldrick, 2008 ▶); program(s) used to refine structure: *SHELXTL*; molecular graphics: *SHELXTL*; software used to prepare material for publication: *SHELXTL* and *PLATON* (Spek, 2003 ▶).

## Supplementary Material

Crystal structure: contains datablocks global, I. DOI: 10.1107/S1600536808035745/ci2702sup1.cif

Structure factors: contains datablocks I. DOI: 10.1107/S1600536808035745/ci2702Isup2.hkl

Additional supplementary materials:  crystallographic information; 3D view; checkCIF report

## Figures and Tables

**Fig. 1.**
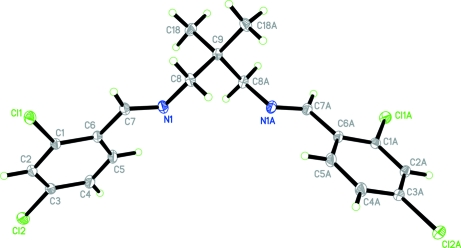
The molecular structure of the title compound, with atom labels and 50% probability ellipsoids for non-H atoms. Atoms labelled with the suffix A are generated by the symmetry operation (1/2 - x, 2 - y, z).

**Fig. 2.**
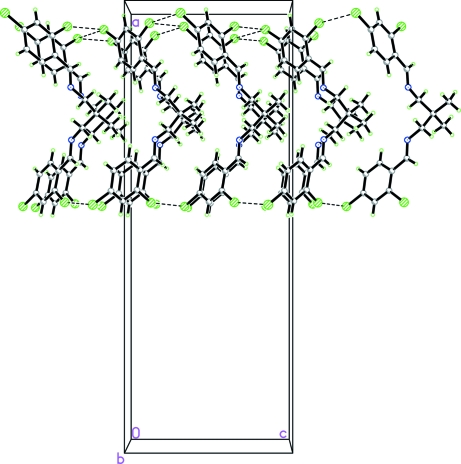
Part of the crystal structure of the title compound, viewed down the b-axis. Dashed lines indicate intermolecular Cl···Cl interactions.

## supplementary crystallographic information

### Comment

Schiff bases are one of most prevalent mixed-donor ligands in the field of coordination chemistry. They play an important role in the development of coordination chemistry related to catalysis and enzymatic reactions, magnetism, and supramolecular architectures. Crystal structures of Schiff bases derived from substituted benzaldehydes and closely related to the title compound have been reported earlier (Li *et al.*, 2005; Bomfim *et al.*, 2005; Glidewell *et al.*, 2005, 2006; Sun *et al.*, 2004; Fun *et al.*, 2008).

The molecule of the title Schiff base compound has crystallographic twofold rotation symmetry (Fig. 1). Bond lengths are within normal ranges (Allen *et al.*, 1987). The plane of the –C═N—C– group is twisted away from the benzene ring by 7.5 (1)°.

In the crystal structure, weak intermolecular Cl···Cl contacts [Cl1···Cl2(*x*,1 - *y*,-1/2 + *z*) = 3.4851 (3) Å] link neighbouring molecules into a two-dimensional network parallel to the *bc* plane (Fig.2).

### Experimental

The synthetic method has been described earlier (Fun *et al.*, 2008). Single crystals suitable for *X*-ray diffraction were obtained by evaporation of an ethanol solution at room temperature.

### Refinement

H atoms were positioned geometrically and refined using a riding model, with C—H = 0.93 Å for aromatic and 0.97 Å for methylene and 0.96 Å for methyl H atoms.

### Crystal data

**Table:**

C_19_H_18_Cl_4_N_2_	*F*_000_ = 856
*M**_r_* = 416.15	*D*_x_ = 1.452 Mg m^−^^3^
Orthorhombic, *P**c**c**a*	Mo *K*α radiation λ = 0.71073 Å
Hall symbol: -P 2a 2ac	Cell parameters from 9929 reflections
*a* = 30.7633 (4) Å	θ = 3.6–41.1º
*b* = 5.4012 (1) Å	µ = 0.63 mm^−^^1^
*c* = 11.4532 (1) Å	*T* = 100.0 (1) K
*V* = 1903.05 (5) Å^3^	Block, colourless
*Z* = 4	0.43 × 0.25 × 0.23 mm

### Data collection

**Table:**

Bruker SMART APEXII CCD area-detector diffractometer	6565 independent reflections
Radiation source: fine-focus sealed tube	5678 reflections with *I* > 2σ(*I*)
Monochromator: graphite	*R*_int_ = 0.028
*T* = 100.0(1) K	θ_max_ = 42.5º
φ and ω scans	θ_min_ = 1.3º
Absorption correction: multi-scan(SADABS; Bruker, 2005)	*h* = −52→57
*T*_min_ = 0.774, *T*_max_ = 0.872	*k* = −9→10
86683 measured reflections	*l* = −20→20

### Refinement

**Table:**

Refinement on *F*^2^	Secondary atom site location: difference Fourier map
Least-squares matrix: full	Hydrogen site location: inferred from neighbouring sites
*R*[*F*^2^ > 2σ(*F*^2^)] = 0.030	H-atom parameters constrained
*wR*(*F*^2^) = 0.099	*w* = 1/[σ^2^(*F*_o_^2^) + (0.0454*P*)^2^ + 0.5965*P*] where *P* = (*F*_o_^2^ + 2*F*_c_^2^)/3
*S* = 1.19	(Δ/σ)_max_ = 0.001
4992 reflections	Δρ_max_ = 0.51 e Å^−^^3^
115 parameters	Δρ_min_ = −0.30 e Å^−^^3^
Primary atom site location: structure-invariant direct methods	Extinction correction: none

### Special details

**Table:**

Experimental. The low-temperature data was collected with the Oxford Cyrosystem Cobra low-temperature attachment.
Geometry. All e.s.d.'s (except the e.s.d. in the dihedral angle between two l.s. planes) are estimated using the full covariance matrix. The cell e.s.d.'s are taken into account individually in the estimation of e.s.d.'s in distances, angles and torsion angles; correlations between e.s.d.'s in cell parameters are only used when they are defined by crystal symmetry. An approximate (isotropic) treatment of cell e.s.d.'s is used for estimating e.s.d.'s involving l.s. planes.
Refinement. Refinement of *F*^2^ against ALL reflections. The weighted *R*-factor *wR* and goodness of fit *S* are based on *F*^2^, conventional *R*-factors *R* are based on *F*, with *F* set to zero for negative *F*^2^. The threshold expression of *F*^2^ > σ(*F*^2^) is used only for calculating *R*-factors(gt) *etc*. and is not relevant to the choice of reflections for refinement. *R*-factors based on *F*^2^ are statistically about twice as large as those based on *F*, and *R*- factors based on ALL data will be even larger.

### Fractional atomic coordinates and isotropic or equivalent isotropic displacement parameters (Å^2^)

**Table:**

	*x*	*y*	*z*	*U*_iso_*/*U*_eq_	
Cl1	0.053693 (7)	1.12278 (4)	0.346996 (17)	0.01671 (5)	
Cl2	0.046757 (8)	0.37440 (4)	0.653936 (18)	0.02040 (6)	
N1	0.19231 (2)	0.96758 (14)	0.31674 (6)	0.01660 (12)	
C1	0.08049 (2)	0.88739 (14)	0.42126 (6)	0.01301 (12)	
C2	0.05625 (3)	0.74765 (14)	0.49989 (6)	0.01467 (12)	
H2A	0.0270	0.7822	0.5127	0.018*	
C3	0.07667 (3)	0.55568 (15)	0.55865 (7)	0.01504 (12)	
C4	0.12041 (3)	0.50138 (16)	0.54095 (7)	0.01807 (13)	
H4A	0.1336	0.3713	0.5807	0.022*	
C5	0.14395 (3)	0.64492 (16)	0.46298 (8)	0.01748 (13)	
H5A	0.1732	0.6098	0.4510	0.021*	
C6	0.12480 (2)	0.84188 (14)	0.40166 (7)	0.01397 (12)	
C7	0.15133 (2)	0.99635 (15)	0.32244 (7)	0.01527 (12)	
H7A	0.1379	1.1157	0.2763	0.018*	
C8	0.21668 (3)	1.13674 (14)	0.24238 (7)	0.01553 (13)	
H8A	0.2317	1.2566	0.2909	0.019*	
H8B	0.1966	1.2262	0.1925	0.019*	
C9	0.2500	1.0000	0.16598 (9)	0.01351 (16)	
C18	0.22667 (3)	0.81059 (16)	0.08866 (7)	0.01774 (13)	
H18A	0.2044	0.8922	0.0443	0.027*	
H18B	0.2138	0.6850	0.1368	0.027*	
H18C	0.2472	0.7358	0.0364	0.027*	

### Atomic displacement parameters (Å^2^)

**Table:**

	*U*^11^	*U*^22^	*U*^33^	*U*^12^	*U*^13^	*U*^23^
Cl1	0.01438 (9)	0.01789 (9)	0.01786 (9)	0.00404 (6)	0.00073 (5)	0.00121 (5)
Cl2	0.02045 (10)	0.02338 (10)	0.01738 (9)	−0.00623 (7)	0.00240 (6)	0.00349 (6)
N1	0.0117 (3)	0.0205 (3)	0.0176 (3)	−0.0015 (2)	0.0016 (2)	0.0025 (2)
C1	0.0116 (3)	0.0143 (3)	0.0131 (3)	0.0006 (2)	0.0001 (2)	−0.0008 (2)
C2	0.0117 (3)	0.0175 (3)	0.0149 (3)	−0.0002 (2)	0.0016 (2)	−0.0010 (2)
C3	0.0143 (3)	0.0170 (3)	0.0139 (3)	−0.0031 (2)	0.0004 (2)	0.0006 (2)
C4	0.0136 (3)	0.0197 (3)	0.0209 (3)	−0.0004 (2)	−0.0016 (2)	0.0046 (3)
C5	0.0108 (3)	0.0207 (3)	0.0209 (3)	0.0005 (2)	−0.0002 (2)	0.0044 (3)
C6	0.0102 (3)	0.0168 (3)	0.0149 (3)	−0.0004 (2)	0.0005 (2)	0.0009 (2)
C7	0.0120 (3)	0.0178 (3)	0.0160 (3)	−0.0004 (2)	0.0010 (2)	0.0015 (2)
C8	0.0120 (3)	0.0163 (3)	0.0183 (3)	−0.0012 (2)	0.0021 (2)	0.0009 (2)
C9	0.0113 (4)	0.0144 (4)	0.0148 (4)	−0.0023 (3)	0.000	0.000
C18	0.0169 (3)	0.0176 (3)	0.0187 (3)	−0.0033 (3)	−0.0036 (2)	−0.0008 (2)

### Geometric parameters (Å, °)

**Table:**

Cl1—C1	1.7376 (8)	C5—H5A	0.93
Cl2—C3	1.7311 (8)	C6—C7	1.4783 (11)
N1—C7	1.2720 (10)	C7—H7A	0.93
N1—C8	1.4566 (10)	C8—C9	1.5369 (10)
C1—C2	1.3917 (11)	C8—H8A	0.97
C1—C6	1.4033 (11)	C8—H8B	0.97
C2—C3	1.3866 (11)	C9—C18	1.5316 (10)
C2—H2A	0.93	C9—C18^i^	1.5316 (10)
C3—C4	1.3917 (11)	C9—C8^i^	1.5369 (10)
C4—C5	1.3869 (12)	C18—H18A	0.96
C4—H4A	0.93	C18—H18B	0.96
C5—C6	1.4043 (11)	C18—H18C	0.96
			
C7—N1—C8	117.61 (7)	N1—C7—H7A	119.7
C2—C1—C6	121.94 (7)	C6—C7—H7A	119.7
C2—C1—Cl1	117.35 (6)	N1—C8—C9	112.00 (6)
C6—C1—Cl1	120.71 (6)	N1—C8—H8A	109.2
C3—C2—C1	118.49 (7)	C9—C8—H8A	109.2
C3—C2—H2A	120.8	N1—C8—H8B	109.2
C1—C2—H2A	120.8	C9—C8—H8B	109.2
C2—C3—C4	121.66 (7)	H8A—C8—H8B	107.9
C2—C3—Cl2	119.21 (6)	C18—C9—C18^i^	109.36 (9)
C4—C3—Cl2	119.12 (6)	C18—C9—C8^i^	108.71 (4)
C5—C4—C3	118.74 (8)	C18^i^—C9—C8^i^	109.73 (4)
C5—C4—H4A	120.6	C18—C9—C8	109.73 (4)
C3—C4—H4A	120.6	C18^i^—C9—C8	108.71 (4)
C4—C5—C6	121.76 (7)	C8^i^—C9—C8	110.59 (9)
C4—C5—H5A	119.1	C9—C18—H18A	109.5
C6—C5—H5A	119.1	C9—C18—H18B	109.5
C1—C6—C5	117.40 (7)	H18A—C18—H18B	109.5
C1—C6—C7	122.39 (7)	C9—C18—H18C	109.5
C5—C6—C7	120.19 (7)	H18A—C18—H18C	109.5
N1—C7—C6	120.65 (7)	H18B—C18—H18C	109.5
			
C6—C1—C2—C3	−1.08 (11)	Cl1—C1—C6—C7	2.99 (11)
Cl1—C1—C2—C3	178.81 (6)	C4—C5—C6—C1	−0.64 (13)
C1—C2—C3—C4	0.20 (12)	C4—C5—C6—C7	177.81 (8)
C1—C2—C3—Cl2	−178.30 (6)	C8—N1—C7—C6	−176.18 (7)
C2—C3—C4—C5	0.42 (13)	C1—C6—C7—N1	172.02 (8)
Cl2—C3—C4—C5	178.92 (7)	C5—C6—C7—N1	−6.36 (12)
C3—C4—C5—C6	−0.19 (13)	C7—N1—C8—C9	−134.46 (8)
C2—C1—C6—C5	1.29 (11)	N1—C8—C9—C18	59.07 (9)
Cl1—C1—C6—C5	−178.59 (6)	N1—C8—C9—C18^i^	178.63 (7)
C2—C1—C6—C7	−177.13 (7)	N1—C8—C9—C8^i^	−60.85 (5)

Symmetry codes: (i) −*x*+1/2, −*y*+2, *z*.
